# Toxic Shock Syndrome Caused by Methicillin-Resistant *Staphylococcus aureus* (MRSA) After Expander-Based Breast Reconstruction

**Published:** 2016-01-08

**Authors:** Hirotaka Suga, Tomohiro Shiraishi, Akihiko Takushima, Kiyonori Harii

**Affiliations:** Department of Plastic Surgery, Kyorin University School of Medicine, Tokyo, Japan

**Keywords:** toxic shock syndrome, breast reconstruction, expander, complication, methicillin-resistant *Staphylococcus aureus*

## Abstract

**Objective:** Toxic shock syndrome is a rare but life-threatening complication after plastic surgery procedures. **Methods:** We experienced 2 cases of toxic shock syndrome after expander-based breast reconstruction caused by methicillin-resistant *Staphylococcus aureus*. **Results:** The first patient took a severe clinical course due to the delayed diagnosis and treatment, and the second patient recovered rapidly after the early diagnosis and treatment based on our experience of the first case. Fever, rash, and gastrointestinal symptoms (diarrhea and/or vomiting) were characteristic and important for the early diagnosis of toxic shock syndrome. **Conclusions:** Considering the increased prevalence of methicillin-resistant *Staphylococcus aureus*, we should suspect methicillin-resistant *Staphylococcus aureus* in cases of toxic shock syndrome that occur postoperatively, and the empiric administration of vancomycin should be initiated in such cases.

Toxic shock syndrome (TSS) is an acute, multisystem, toxin-mediated illness, often resulting in multiorgan failure.^1^ It is caused by toxin-producing strains of *Staphylococcus aureus* and *Streptococcus pyogenes*. Since its first report by Todd et al,^2^ TSS has been reported in a variety of clinical situations including vaginal infections, burns, and surgical wound infections.^3^ In the field of plastic surgery, too, TSS has been recognized as a rare but life-threatening complication.^4^^,^^5^ In particular, several cases of TSS associated with breast implants have been reported.^6^^-^9 Here, we report 2 cases of TSS after expander-based breast reconstruction caused by methicillin-resistant *Staphylococcus aureus* (MRSA).

## METHODS

**Figure 1:**
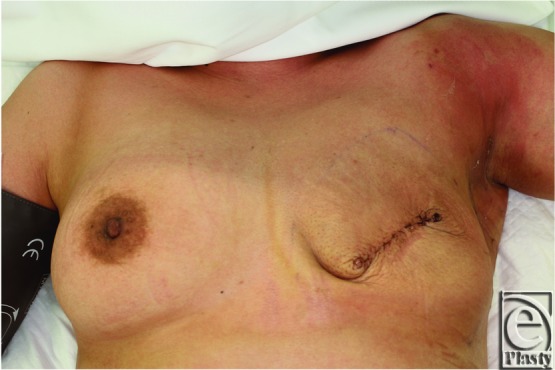
Case 1 on the first day of onset. She presented with a diffuse rash on her upper extremities, while the site of operation (left breast) showed no symptoms.

Case 1 was a 40-year-old woman, and she underwent a mastectomy and sentinel node biopsy for left breast cancer. Immediately after the surgery, a tissue expander was implanted under the pectoralis major muscle for breast reconstruction. Prophylactic antibiotics (piperacillin, 4 g per day) were administered for 9 days following the surgery, until all drains were removed. On postoperative day 10, the patient presented with a fever of 39.3°C, a diffuse rash on her upper extremities, hypotension, and diarrhea (Fig 1). The site of operation showed no drainage or erythema. We first suspected that the patient's symptoms were due to a viral infection or allergic reaction. Her general condition worsened, and she was admitted to the intensive care unit.

**Figure 2:**
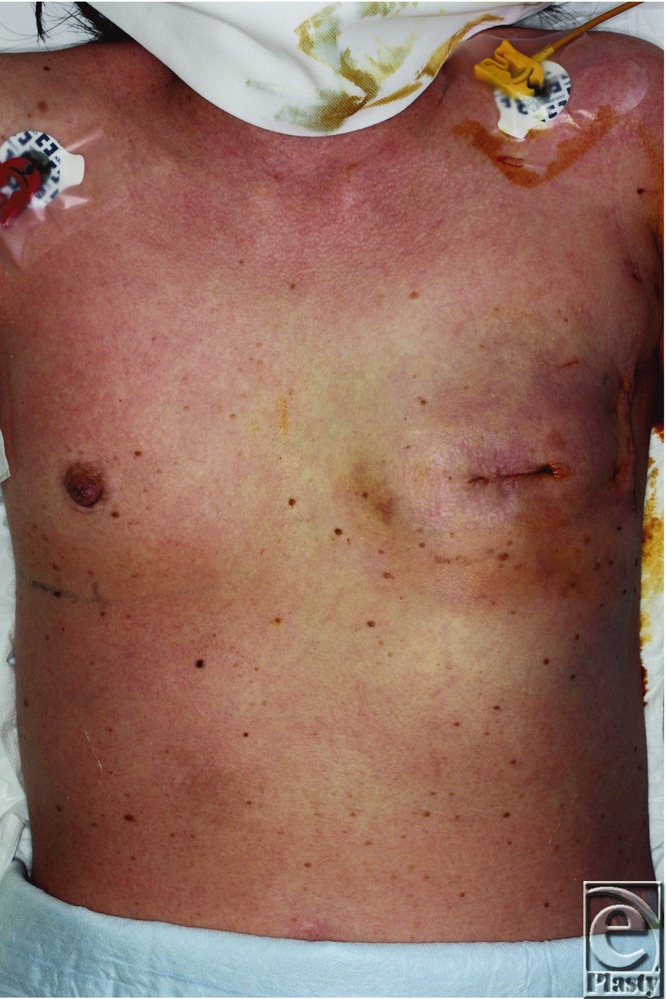
Case 2 on the first day of onset. She presented with a diffuse rash on the upper part of her body.

Case 2 was a 54-year-old woman, and she underwent a mastectomy and sentinel node biopsy for left breast cancer. Immediately after the surgery, a tissue expander was implanted under the pectoralis major muscle for breast reconstruction. Prophylactic antibiotics (cefazolin, 2 g per day) were administered for 7 days following the surgery, until all drains were removed. On postoperative day 8, the patient presented with a fever of 40.0°C, a diffuse rash on the upper part of her body, hypotension, and vomiting (Fig 2).

## RESULTS

**Figure 3:**
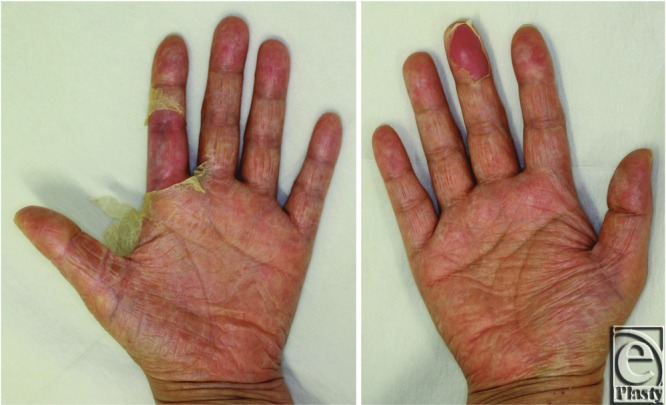
The hands of case 1 on the ninth day of onset. The desquamation was first noted on the fifth day.

**Figure 4:**
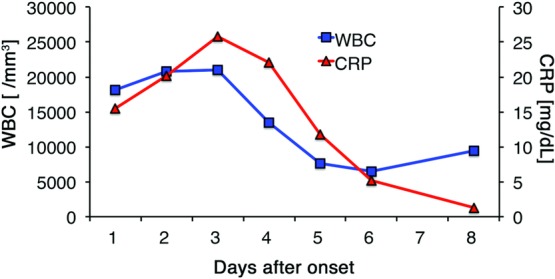
Time course of laboratory data, WBC, and CRP in case 1. CRP indicates C-reactive protein; WBC, white blood cell.

In case 1, laboratory investigations revealed a white blood cell (WBC) count of 18,100/mm^3^ (reference range, 3,300–8,600/mm^3^) and a C-reactive protein (CRP) level at 15.5 mg/dL (reference range, 0–0.14 mg/dL). The data showed renal dysfunction, with 33.9 mg/dL of blood urea nitrogen (reference range, 8.0–20.0 mg/dL) and creatinine at 3.11 mg/dL (reference range, 0.46–0.79 mg/dL). The data also showed liver dysfunction, with total bilirubin at 3.0 mg/dL (reference range, 0.4–1.5 mg/dL), lactate dehydrogenase at 272 IU/L (reference range, 124–222 IU/L), aspartate transaminase at 84 IU/L (reference range, 13–30 IU/L), and alanine transaminase at 212 IU/L (reference range, 7–23 IU/L). Dopamine and noradrenaline were administered to maintain the patient's blood pressure. Late at night on the day of onset, we diagnosed the patient's condition as TSS and removed the tissue expander under local anesthesia. The patient was cared for in the intensive care unit for 4 days, and dopamine and noradrenaline were continued during that period. Broad-spectrum antibiotics (meropenem, 1.5 g per day) were first administered for 2 days and were changed to vancomycin (2 g per day) when intraoperative wound cultures revealed MRSA. On the fifth day after the onset of TSS, the patient presented with desquamation of the hands (Fig 3). Her condition gradually improved, and she was discharged home on the 10th day after the onset of TSS (Fig 4).

**Figure 5:**
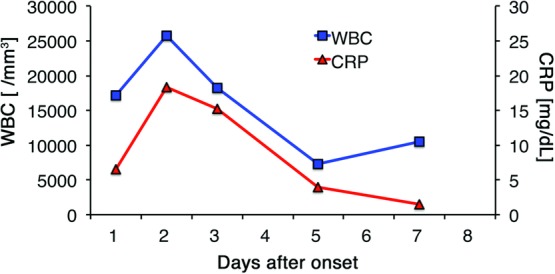
Time course of laboratory data, WBC, and CRP, in case 2. CRP indicates C-reactive protein; WBC, white blood cell.

In case 2, laboratory data revealed WBC count of 17,200/mm^3^ (reference range, 3,300–8,600/mm^3^) and CRP level at 6.5 mg/dL (reference range, 0–0.14 mg/dL). The data showed liver dysfunction with lactate dehydrogenase at 336 IU/L (reference range, 124–222 IU/L), aspartate transaminase at 189 IU/L (reference range, 13–30 IU/L), and alanine transaminase at 81 IU/L (reference range, 7–23 IU/L). On the basis of our experience with case 1, we immediately diagnosed this patient's condition as TSS. The tissue expander was removed 3 hours after the onset of TSS. Vancomycin (2 g per day) was started on the first day of onset in addition to a broad-spectrum antibiotic (meropenem, 1.5 g per day). The patient's condition rapidly improved, and she was discharged home on the ninth day after the onset of TSS (Fig 5). Intraoperative wound cultures grew MRSA. The patient presented with slight desquamation on the hands.

## DISCUSSION

In case 1, the diagnosis of TSS was delayed because the symptoms of infection at the site of operation were mild and other symptoms (fever, rash, hypotension, and diarrhea) were attributed to a viral infection or allergic reaction. Vancomycin was not used until the intraoperative wound cultures revealed MRSA. We believe that the delay of diagnosis and treatment in case 1 caused the delayed recovery of the patient even after the removal of the tissue expander. In case 2, an early diagnosis of TSS was made on the basis of our experience with case 1. The tissue expander was removed soon after the onset, and vancomycin was started on the first day. The early diagnosis and treatment in case 2 led to her rapid recovery.

The case definition of TSS according to the criteria of the US Centers for Disease Control and Prevention includes fever, rash, desquamation, and hypotension, in addition to the presentation of specific abnormalities in at least 3 specified organ systems (gastrointestinal, muscular, mucous membrane, renal, hepatic, hematologic, and central nervous system).^8^ However, desquamation is not observed at the time of onset and usually occurs 1 week after onset. Abnormalities in organ systems vary among patients and depend on the severity of the individual patient's condition. Among the symptoms presented in TSS, we believe that fever, rash, and gastrointestinal abnormalities (diarrhea and/or vomiting) are characteristic and important for early diagnosis. In fact, these symptoms were presented in most of the previous cases,^5^^-^9 as well as in our 2 cases.

MRSA strains have increased in prevalence during the last decade, and MRSA has been reported as a cause of TSS.^10^ Particularly in surgical wound infections, prophylactic antibiotics are usually administered perioperatively, and MRSA is more likely to be the cause of TSS in such scenarios. We believe that in cases of TSS that occur postoperatively, MRSA should be suspected and an empiric administration of vancomycin should be initiated.

In conclusion, although it is a rare postoperative complication, all plastic surgeons should be aware of TSS. The early diagnosis of TSS based on its characteristic symptoms of fever, rash, and gastrointestinal abnormalities and its prompt treatment are crucial.
